# Supplementary material for: Getting new algorithmic results by extending distance-hereditary graphs via split composition
Source: PeerJ Comput Sci. 2021 Jul 7;7:e627. doi: 10.7717/peerj-cs.627 (PMC8279144; doi:10.7717/peerj-cs.627)
Supplement: Supplemental Information 1 — Algorithm 2 decomposes G according to the Cunnigham decomposition, chooses any component B as root, and call the recursive Algorithm 1 by passing it the root B. The final result is the stretch number s(G). [file peerj-cs-07-627-s001.pdf]

Algorithm 1: function $Z(T, B)$, $T = T(G)$, for $G \in \mathrm{sDH}(2)$

Input: $T(G)$ is assumed rooted, B is a component of $T(G)$
Output: the pair $[\zeta(\bar{B}),\ \zeta_{\bar{B}}(m)]$

Let $\{B_1, B_2, \ldots, B_k\}$ the children of B connected to B by edges (m_i, m_i'), $i = 1, \ldots, k$, where $m_i \in B$
Let m the marked vertex connecting B to its parent in T, if it exists.
Let $a = b = c = d = e = f = \langle 0, 0\rangle$
$a = \max\{\zeta(u, v) \mid (u, v)$ is a stretch-pair of $B\}$
$b = \max\{\mathit{first}(Z(T, B_i)) \mid i = 1, \ldots, k\}$
$c = \max\{\zeta_B(m_i) + \mathit{second}(Z(T, B_i)) \mid i = 1, \ldots, k\}$
$d = \max\{\zeta(m_i, m_j) + \mathit{second}(Z(T, B_i)) + \mathit{second}(Z(T, B_j)) \mid (m_i, m_j)$ is a stretch-pair of $B\}$
if *m exists* **then**
    $e = \zeta_B(m)$
    $f = \max\{\zeta(m, m_i) + \mathit{second}(Z(T, B_i)) \mid (m, m_i)$ is a stretch-pair of $B\}$
return $[\max\{a, b, c, d\},\ \max\{e, f\}]$

Algorithm 2: computing $s(G)$

Input: a graph G with any component B of $\mathcal{D}(G)$ such that $s(B) < 2$
Output: $s(G)$

compute $\mathcal{D}(G)$ and $T(G)$
choose a component B of $\mathcal{D}(G)$ as a root of $T(G)$
return $\sigma(\mathit{first}(Z(T(G), B)))$
